# Supplementary material for: Clorfl86/RHEX Is a Negative Regulator of SCF/KIT Signaling in Human Skin Mast Cells
Source: Cells. 2023 May 3;12(9):1306. doi: 10.3390/cells12091306 (PMC10177086; doi:10.3390/cells12091306)
Supplement: Supplementary file 1 [file cells-12-01306-s001.zip › cells-2284042-supplementary.pdf]

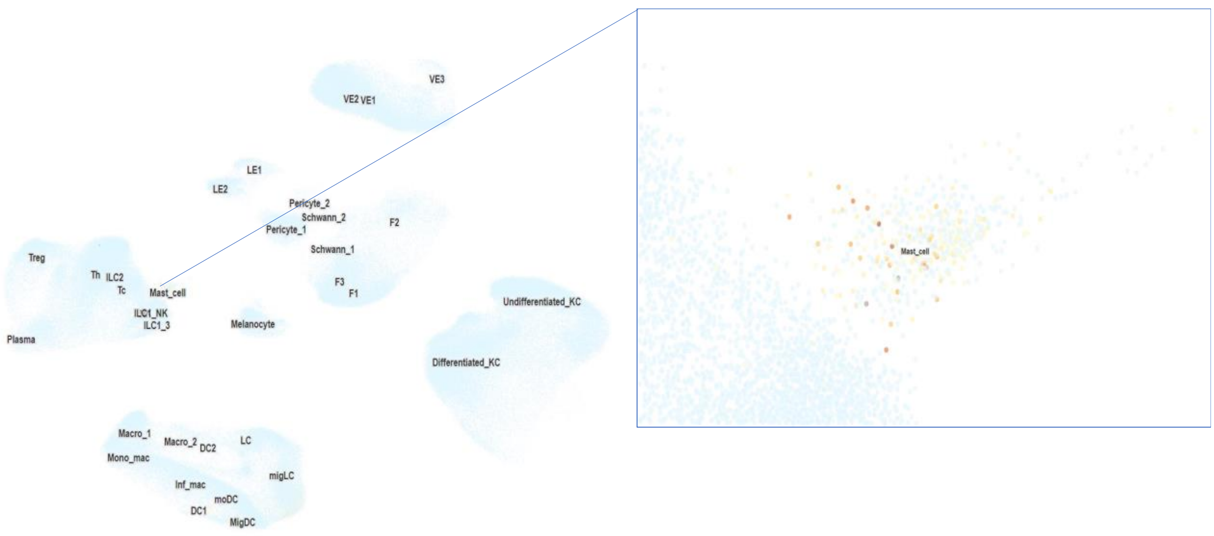

Figure S1: Larger version of Figure 1b, upper part. Note that the brown staining highlighting RHEX expression is almost exclusively found in clusters identifying MC.

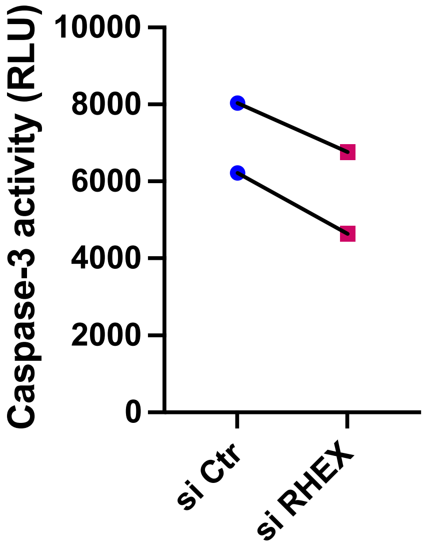

Figure S2: RHEX is a proapoptotic factor in SCF-activated skin MCs as its knockdown leads to attenuated caspase-3 activity. Caspase-3 activity was determined by the Caspase-Glo 3/7 assay 24 h after siRNA-initiated knockdown (si RHEX) against control (si ctrl). Data of two individual cultures are shown as interconnected dots RLU=Relative Luminescence Units.

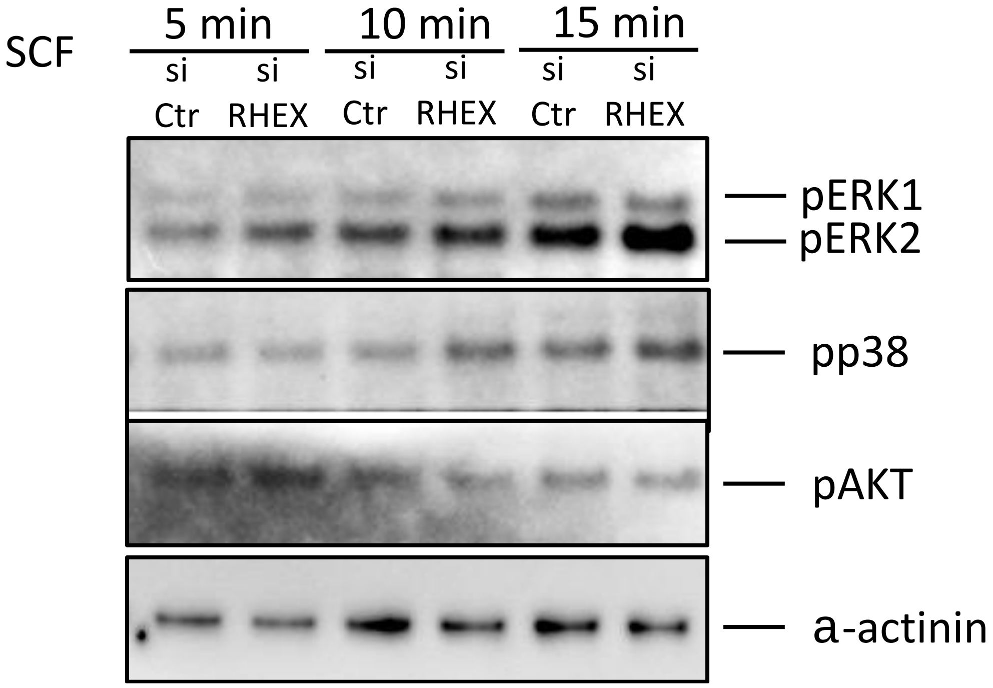

Figure S3: ERK1/2 and p38 phosphorylation is constrained by RHEX at different time points after SCF addition. RHEX silencing was achieved by exposing skin MCs to RHEX-siRNA (si RHEX) for 2 d against control (si ctr) as in Figures 2 and 3. Upon silencing, cells were stimulated with SCF (100 ng/ml) for 5, 10 and 15 min, and signaling components were detected by immunoblotting. Detection of the distinct proteins was performed consecutively on the same membrane. α-actinin served as the loading control. One of two independent experiments with comparable outcome is shown.

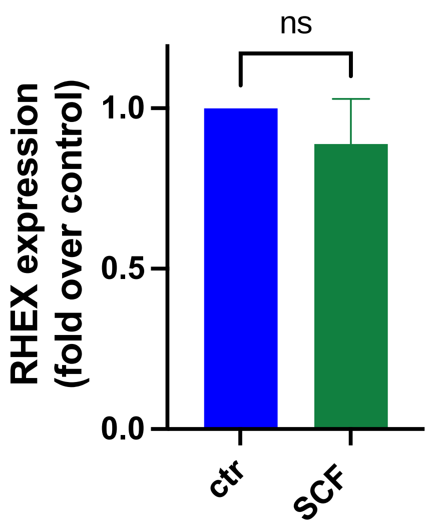

Figure S4: RHEX expression remains unaffected by SCF in skin MCs. Skin MCs (deprived of growth factors overnight) were treated wit/out SCF (at 100 ng/ml) for 4 h and harvested for RNA extraction. RHEX expression was determined by RT-qPCR and normalized as described in Methods. Expression in SCF-stimulated cells is given relative to the matching control of the same experiment (culture). Mean ± SEM of 7 independent experiments. Ctr - control

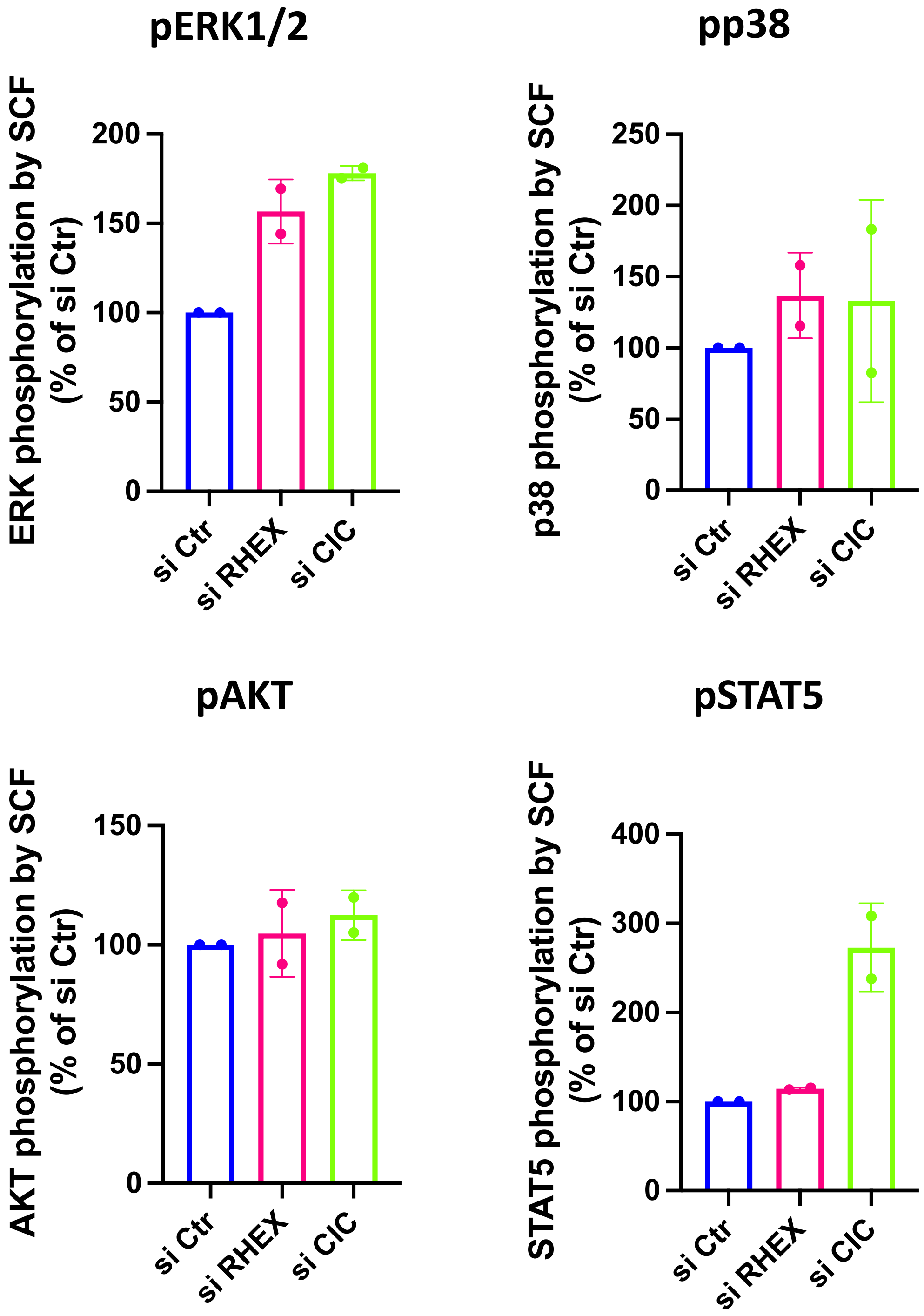

Figure S5: CIC potently interferes with STAT5 phosphorylation, while RHEX does not. Signaling components were detected by immunoblotting and semi-quantified by ImageJ. Corresponds to main Figure 4. Please note that while differences in pERK and pp38 against Ctr (control) cannot be accurately judged from only two blots, the impact of si CIC is striking in case of pSTAT5 (and not reproduced by si RHEX).
